# Estimation of Value-Based Price for 48 High-Technology Medical Devices

**DOI:** 10.7759/cureus.39934

**Published:** 2023-06-04

**Authors:** Giulia Hyeraci, Sabrina Trippoli, Melania Rivano, Andrea Messori

**Affiliations:** 1 Health Sciences, Agenzia Regionale Toscana, Firenze, ITA; 2 Health Technology Assessment (HTA) Unit, Regione Toscana, Firenze, ITA; 3 Hospital Pharmacy, Binaghi Hospital, Cagliari, ITA

**Keywords:** medicine pricing, value-based pricing, pricing, cost-effectiveness analysis, cost-effectiveness, health care outcomes, value in health care

## Abstract

Value-based price is estimated quite frequently for medicines, but its application to medical devices is scarce. While some reports have been published in which this parameter has occasionally been determined for devices, no large-scale application has yet been reported. Our objective was to pursue a systematic analysis of the literature published on value-based prices of medical devices. Pertinent papers were selected upon the criterion that the value-based price was reported for the device examined. The real prices of the devices were compared with their values of value-based price and the ratios between real price versus value-based price were calculated. A total of 239 economic articles focused on high-technology medical devices were selected from a standard PubMed search. Among these, the proportion of analyses unsuitable for value-based price estimation was high (191/239; 80%), whereas adequate clinical and economic information for estimating this parameter was available in 48 cases (20%). Standard equations of cost-effectiveness were applied. The value-based price was determined according to a willingness-to-pay threshold of 60,000 € per quality-adjusted life year. Real prices of devices were compared with the corresponding estimates of value-based prices. From each analysis, we extracted also the value of incremental cost-effectiveness ratio (ICER). Our final dataset included 47 analyses because one was published twice. There were five analyses in which the ICER could be estimated for the treatment, but not for the device. In the dataset of 42 analyses with complete information, 36 out of 42 devices (86%) were found to have an ICER lower than the pre-specified threshold (favorable ICER). Three ICERs were borderline. A separate analysis was conducted on the other three devices that showed an ICER substantially greater than the threshold (unfavorable ICER). Regarding value-based prices, the values of real price were appreciably lower than the corresponding value-based price in 36 cases (86%). For three devices, the real price was substantially higher than the value-based price. In the remaining three cases, real prices and value-based prices were very similar. To our knowledge, this is the first experience in which a systematic analysis of the literature has been focused on the application of value-based pricing in the field of high-technology devices. Our results are encouraging and suggest a wider application of cost-effectiveness in this field.

## Introduction and background

Value-based pricing represents an approach for managing healthcare products in which the price of the product is assessed according to cost-effectiveness; this implies that the cost-effectiveness ratio of the product is determined and compared with the current standard of willingness-to-pay (WTP) threshold. This concept has been debated in the scientific literature for more than 20 years, and its application has generally been focused on medicines, particularly in the area of innovative agents [1-7]. A number of national regulatory agencies (e.g., in the United Kingdom, France, Germany, and Canada) have formally adopted algorithms that estimate the value-based price for newly introduced medicines [2,7,8]. To our knowledge, no reports can be found in the literature published thus far that describe any systematic analysis of value-based pricing in the field of medical devices [8-12]. A PubMed search conducted from inception to the present date shows a series of sporadic reports of value-based pricing applied to individual devices, particularly high-technology ones; among these, numerous reports reflect a research project sponsored by the manufacturer [8-12].

In a previous article [12], we analyzed a small group of high-technology devices that were consecutively approved in the Tuscany region from January 2020 to December 2021 [13]. In the present work, we describe a more extensive analysis based on literature data and focused on the experiences of value-based pricing published thus far in the field of medical devices. In these studies, the value-based prices were computed according to standard cost-effectiveness methods and were also compared with the current prices obtained from the Italian market (“real prices”).

## Review

Literature search

A standard PubMed search was conducted to identify medical devices, that had been the subject of an economic analysis (keywords: medical devices, economic analysis, cost-effectiveness analysis, cost-utility analysis, quality-adjusted life year*, QALY*). In general, to apply the method of value-based pricing, a cost-effectiveness model is needed in which both clinical effectiveness and healthcare cost incurred by the device are interpreted according to standard principles of health technology assessment; this approach requires that the clinical effectiveness is expressed in quality-adjusted life years (QALYs). We accepted such models from studies published in the PubMed database provided that the model was judged to be appropriate by the four authors of this report and a specific reference to a peer-reviewed article was available. Given that PubMed was the source of all eligible articles, a minimum standard, a scientific quality was ensured by the peer-review process to which all PubMed articles are subjected; to go beyond this basic criterion, the four authors were requested to confirm the adequacy of the economic model employed in the article by examination of full texts.

Estimation of value-based price

In comparing two hypothetical treatments denoted as A (novel treatment) and B (comparator or standard of care), the standard formula to determine the incremental cost-effectiveness ratio (ICER) is as follows:

ICER = (cost_A_ - cost_B)_ / (QALYs_A_- QALYs_B_) [Equation 1]

where QALYs are quality-adjusted life years per patient and costs are normalized to a patient as well.

In the estimation of value-based price for A, cost_A_ can firstly be split into the price of the device (price_A_) plus the other costs incurred in the clinical use of A (denoted as othercosts_A_). Hence,

cost_A _= price_A_+ othercosts_A_ [Equation 2]

It should be noted that also cost_B_ includes the same two components (i.e., price_B_+ othercosts_B_); however, splitting cost_B_ is not needed if, as in the present case, the calculation is aimed at estimating the value-based price for A. Furthermore, the original analyses are likely to frequently exclude both othercosts_A_ and othercosts_B_ when their two values are identical.

Since ICER is ICER = (price_A_+ othercosts_A_ - cost_B_) / gain_QALYs_ and incrementalcost_AvsB_ is incrementalcost_AvsB_ = price_A_+ othercosts_A_ - cost_B_, the equation of ICER can be rewritten as follows:

ICER = incrementalcost_AvsB_ / gain_QALYs_ [Equation 3]

where the difference QALYs_A_- QALYs_B_ has been denoted as gain_QALYs_.

Finally, the relationship between ICER and incrementalcost_AvsB_ is:

incrementalcost_AvsB_ = ICER x gain_QALYs_ [Equation 4]

and consequently

price_A_+ othercosts_A_ - cost_B_ = ICER x gain_QALYs_ [Equation 5]

and finally

price_A _= ICER x gain_QALYs_ + cost_B_ - othercosts_A _[Equation 6]

If one replaces ICER with the societal value of the willingness to pay threshold (WTP_threshold_), the value-based price for A (denoted as valuebasedprice_A_) can be estimated as follows:

valuebasedprice_A_ = WTP_threshold _x gain_QALYs _+ cost_B_ - othercosts_A _[Equation 7]

Data sources and selection of devices to be included in our analysis

A simple flowchart (not reported herein) was employed to describe the process by which we verified the presence of a suitable cost-effectiveness model. Regarding the currency used in our analysis, our results were expressed in €; different currencies were converted into € according to the Oanda website (http://www.oanda.com/currency-converter/). In our analysis, Equation 7 was used to estimate value-based prices, while the values of gain_QALYs_, cost_A,_ othercosts_A_, and cost_B_ were drawn from the referenced papers; the value of the WTP_threshold_ was set at 60,000 €/QALY gained.

Analysis of selected articles

**Figure 1:**
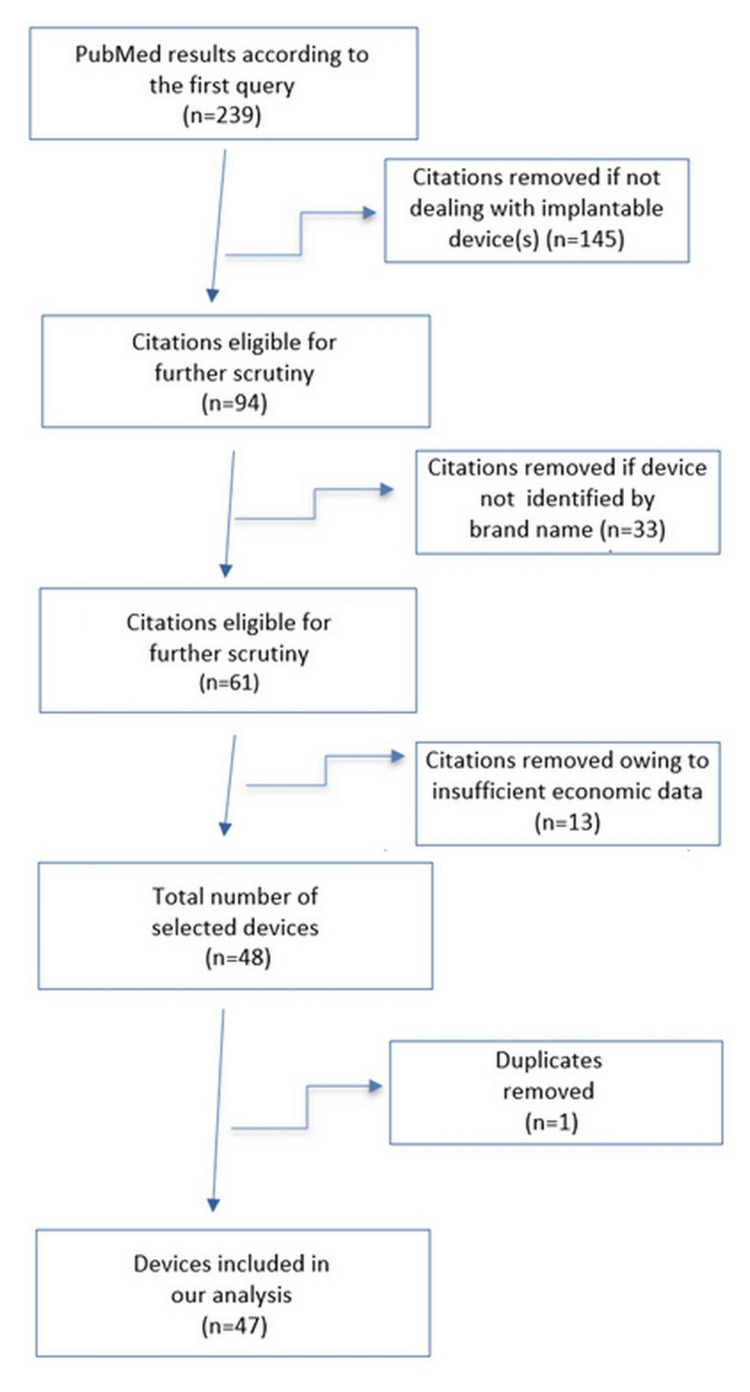
Flow diagram describing our selection of included devices. The first PubMed query was based on the following keywords: “medical devices, economic analysis, cost-effectiveness analysis, cost-utility analysis, quality-adjusted life year*, QALY*) with filter set to last 5 years.

Figure 1 illustrates the flowchart of the selection process that, among the 239 eligible citations, allowed us to select those supported by adequate cost-effectiveness data. Each citation was associated to the device assessed in the analysis; thereafter, our reports presented all results in terms of devices rather than citations. The proportion of analyses unsuitable for value-based price estimation was high (191/239; 80%), whereas adequate clinical and economic information was available in 48 cases (20%). Our final analysis involved 47 devices because one duplicate study was found in our literature search. Of these, 20 referred to the cardiological area, while the remaining 27 referred to other clinical disciplines.

Table 1 (in columns 1 through 9) shows the main information about these 48 devices (including the duplicate study) along with the respective sources of cost-effectiveness data. In the dataset of 42 analyses with complete information, four devices (9.5%) were found to have an ICER substantially higher than the threshold; three (7.1%) had a borderline ICER (i.e., ±15% compared with the threshold of 60,000 €/QALY), while the remaining 35 (83.3%) had an ICER clearly lower than the threshold. These values represent the ICER based on the device (not based on the treatment).

**Table 1:** Detailed information (real price, value-based price, and their ratio) about the devices examined in the 48 economic analyses. CARDIO, device employed in cardiology; CEA, cost-effectiveness analysis; QALY, quality-adjusted life year; ICER, incremental cost-effectiveness ratio; VBP, value-based price. Exchange rates versus €: GBP, 0.833; Canadian $, 0.7463; Norwegian crown, 0.0956.

Area	Device	Indication	First author	Currency used in CEA	Gain in QALYs	Treatment cost in local currency for experimental group	Device cost in local currency	Treatment cost in local currency for controls	Value-based price in € for device	Value-based price in € for treatment	ICER in €/QALY	Value-based price in local currency for the device	Value-based price in local currency for the treatment
CARDIO	Amplatzer	Patent foramen ovale closure	Allou [14]	€	0.16	13877	3735	6576	6034	16176	45631	NA	NA
CARDIO	Barostim neo	Heart failure NYHA class III with LVEF <35%	Borisenko [15]	€	1.19	50856	21000	17671	59215	89071	27887	NA	NA
CARDIO	HEARTMATE 3	End-stage heart failure	Lim [16]	GBP	2.3976	141598	80000	28047	91884	143195	39451	110305	171903
CARDIO	LVAD (left ventricular assist device)	End-stage heart failure	Chew [17]	Canadian$	1.08	286942	NA	31984	NA	72230	NA	NA	96784
CARDIO	Mitraclip	Mitral valve regurgitation	Shore [18]	GBP	1.07	42971	16500	10704	40344	62395	25120	48433	74904
CARDIO	Perceval, suture-less valve	Severe aortic stenosis	Desser [19]	Norwegian crown	0.11	453869	32500	650529	22539	62822	-170915	235760	657129
CARDIO	Propaten	Peripheral Artery Disease	Villemoes [20]	€	0.06	34446	NA	33856	NA	37456	9833	NA	NA
CARDIO	Reducer	Relief of angina symptoms in refractory angina	Gallone [21]	€	0.138	15702	7000	6988	6566	15268	63145	NA	NA
CARDIO	Sapien	Aortic stenosis in patients with intermediate surgical risk	Goodall [22]	€	0.41	34157	NA	54161	NA	75761	NA	NA	NA
CARDIO	SAPIEN 3	Aortic valve stenosis in patients with low-surgical risk	Mennini [23]	€	1.11	42587	31794	39269	95076	105869	2989	NA	NA
CARDIO	SAPIEN 3	Inoperable patients with symptomatic severe aortic stenosis	Pinar [24]	€	1.31	46898	24078	33931	89711	112531	9898	NA	NA
CARDIO	SAPIEN 3	Symptomatic severe aortic stenosis in patients with high surgical risk	Pinar [24]	€	0.44	50950	24078	47413	46941	73813	8039	NA	NA
CARDIO	SAPIEN 3	Symptomatic severe aortic stenosis in patients with intermediate surgical risk	Pinar [24]	€	0.39	49346	24078	47191	45323	70591	5525	NA	NA
CARDIO	SAPIEN 3	Relief of severe aortic stenosis (high surgical risk)	Gilard [25]	€	0.89	38992	15419	51734	81561	105134	14317	NA	NA
CARDIO	SAPIEN 3	Relief of severe aortic stenosis (intermediate surgical risk)	Tarride [26]	Canadian$	0.48	70556	25000	57083	30096	64094	20948	40237	85883
CARDIO	SAPIEN 3	Relief of severe aortic stenosis (high surgical risk)	Tarride [26]	Canadian$	0.43	84348	25000	76986	32417	76709	12777	43438	102786
CARDIO	SU-AVR (sutureless aortic valve replacement)	Aortic stenosis in low surgical risk patients	Povero [27]	€	1.14	26679	6000	33250	80971	101650	-5764	NA	NA
CARDIO	TAVR (transcatheter aortic valve replacement)	Aortic valve replacement in severe aortic stenosis	Tam [28]	Canadian$	0.14	44299	22000	32994	14251	30892	60654	19095	41394
CARDIO	TAVR (transcatheter aortic valve replacement)	Aortic stenosis	Tam [28]	Canadian$	0.23	46904	24000	36356	20338	37431	34226	27252	50156
CARDIO	Tyrx	Aortic stenosis	Kay [29]	GBP	0.03	31261	719	31010	1889	27331	6969	2268	32810
OTHER	Gold Anchor	Targeting radiotherapy by implanting 22G fiducial markers	Lundqvist [30]	SEK	0.015	679	confidential	688	NA	152	NA	NA	1588
OTHER	Y-90 microspheres	Patients with hepatocellular carcinoma ineligible for transarterial chemoembolization	Muszbek [31]	GBP	0.601	29530	8000	30927	37866	55800	-1936	45457	66987
OTHER	Yttrium-90 resin microspheres for transarterial radioembolization	Locally advanced and inoperable hepatocellular carcinoma	Zarca [32]	€	0.006	44345	32534	27166	15715	27526	2863167	NA	NA
OTHER	iStent Inject trabecular bypass stent (TBS) device	Mild-to-moderate open-angle glaucoma in Italy	Ahmed [33]	Canadian$	0.023	21384	1674	21773	2569	17279	12622	3443	23153
OTHER	IStent inject device	Non-infectious intermediate uveitis, posterior uveitis and panuveitis	Fea [34]	€	0.095	8368.51	1300	7134	5766	12835	12995	NA	NA
OTHER	Argus II	Reduced infections after pacemaker or defibrillator implant	Health Quality Ontario [35]	Canadian$	2.5688	537734	179850	287458	62467	329556	72712	83702	441586
OTHER	OZURDEX	Diabetic macular oedema	Pesonen [36]	€	0.08	9810	1075	5563	1628	10363	53088	NA	NA
OTHER	ILUVIEN (Fac implant vs usual care)	Retinitis pigmentosa in pseudo-phakic pts	Pochopien [37]	GBP	0.19	22117	7982	19051	13591	23566	13442	16316	30451
OTHER	ILUVIEN (Fac implant vs DEXA implant)	Diabetic macular oedema in phakic pts	Pochopien [37]	GBP	0.71	24425	8078	20340	38812	52429	4793	46593	62940
OTHER	ILUVIEN (Fac implant vs usual care)	Diabetic macular oedema in phakic pts	Pochopien [37]	GBP	0.11	24425	8078	21255	9586	23203	24005	11508	27855
OTHER	Ozurdex (dexamethasone implant)	Non-infectious uveitis	Squires [38]	GPB	This analysis is the same as that published by Squires et al. (coded as S.38).								
OTHER	DEX-700, dexa- methasone implant (implant + current practice vs current practice alone)	Diabetic macular oedema	Squires [38]	GBP	0.029	40565	870	39992	1698	34763	16459	2037	41732
OTHER	OPRA (Osteointegrated Prostheses for the Rehabilitation of Amputees)	Trans-femoral amputation	Hansson [39]	€	0.28	78417	48139	6880	-6598	23680	255489	NA	NA
OTHER	InSpace	Irreparable rotator cuff tears	Castagna [40]	€	0.05	17327	15000	16805	17478	19805	10440	NA	NA
OTHER	InSpace	Irreparable rotator cuff tears	Castagna [40]	€	0.06	17327	15000	24312	25585	27912	-116417	NA	NA
OTHER	InSpace	Irreparable rotator cuff tears	Castagna [40]	€	0.1	17327	15000	31031	34704	37031	-137040	NA	NA
OTHER	Pathello-femoral arthroplasty (Avon implant by Stryker Orthopaedics, Mahwah, New Jersey, USA)	Pathello-femoral arthroplasty in knee osteoarthritis	Fredborg [41]	€	0.056	6539.5	1603	6867.6	5921	10228	-5859	NA	NA
OTHER	Triangular titanium implant for sacroiliac joint fusion	Patients subjected to minimally invasive sacroiliac joint fusion surgery	Blisset [42]	GBP	0.75	8358	3465	6880	39140	43216	1642	46987	51880
OTHER	aMACE	Revision implant of acetabular arthroplasty	Tack [43]	€	0.05	26559	8419	27824	12684	30824	-25300	NA	NA
OTHER	Biodesign Surgisis (Cook)	Trans-sphincteric fistula-in-ano	Jayne [44]	GBP	0.026	2750	780	2297	1572	3213	14513	1887	3857
OTHER	FENIX TORAX	Treatment of adult fecal incontinence	Jayne [45]	GBP	0.01	11360	4000	10913	3459	9590	37235	4153	11513
OTHER	AUS (artificial urinary sphincter)	Severe post-prostatectomy stress urinary incontinence (PPSUI)	Shamout [46]	Canadian$	1.15	14228	8500	18938	61353	65628	-3057	82210	87938
OTHER	AUS (artificial urinary sphincter)	Severe post-prostatectomy stress urinary incontinence (PPSUI)	Shamout [46]	Canadian$	1.15	14228	8500	18938	61353	65628	-3057	82210	87938
OTHER	OPTOGENERAPY (Optoferon)	Relapsing-remitting multiple sclerosis	Visser [47]	€	0.45	153621	30395	691	-95535	27691	339844	NA	NA
OTHER	Inspire	Obstructive sleep apnea	Pietzsch [48]	€	1.02	99,357	NA	54825	NA	116025	NA	NA	NA
OTHER	Propel (bioabsorbable corticosteroid-eluting sinus implant)	Chronic rhinosinusitis	Javanbakht [49]	GBP	0.001	4646	580	4655	541	3928	-7497	649	4715
OTHER	Nexplanon (etonogestrel 68 mg implant)	Prevention of pregnancy	CADTH [50]	Canadian$	0.00851	768	369.30	691	599	897	6753	803	1202
OTHER	WavelinQ (incident patients)	Endovascular arteriovenous fistula in hemodialysis patients	Rognoni [51]	€	0.018	5722	2544	33041	30943	34121	-1517722	NA	NA

**Figure 2:**
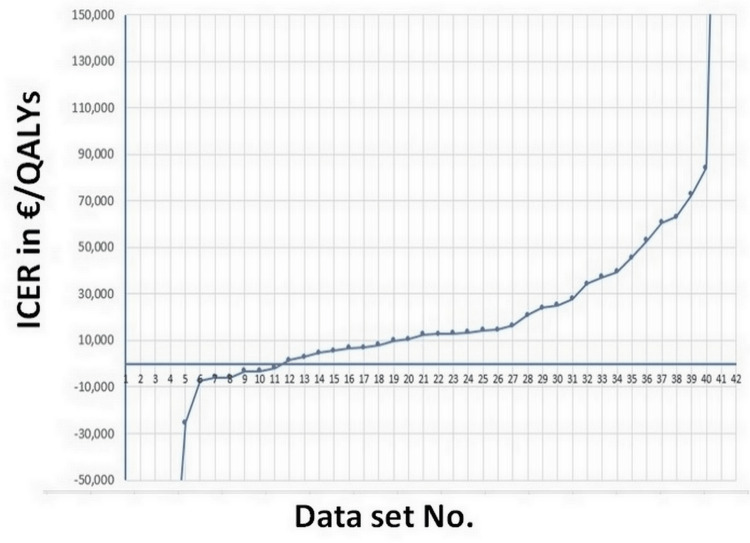
Values of ICER for the 42 analyses ordered from the lowest to the highest. ICER, Incremental cost-effectiveness ratio

Figure 2 summarizes these 42 values of ICER. There were 11 cases in which the ICER was negative because the therapeutic option based on the novel device was dominant.

**Figure 3:**
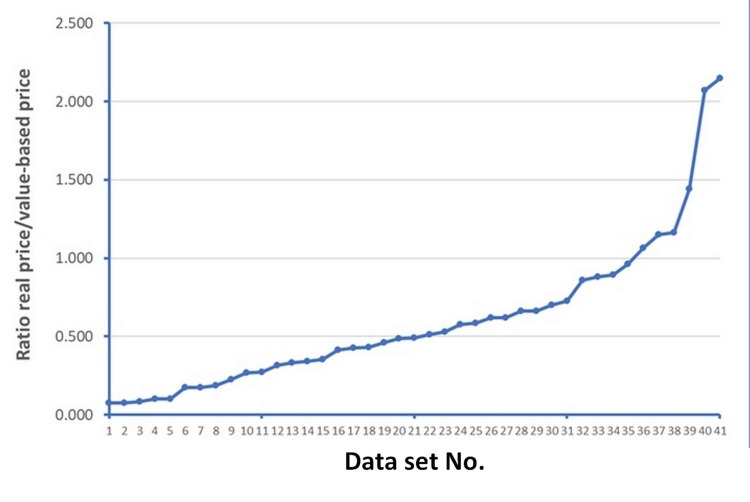
Comparison between real price and value-based price for the 42 devices included in the main analysis. Detailed information on this analysis is presented in Table 2.

Using these cost-effectiveness data, we estimated the values of value-based prices already shown in Table 1 (columns 10, 11, 12, and 14), Figure 3, and Table 2. The calculation of value-based prices was applied separately to the treatment (i.e., all healthcare procedural costs including the device) and to the device. In this framework, the value-based price with respect to the treatment (last column in Table 1) could be estimated in all 47 treatments. On the other hand, the value-based price with respect to the device could not be estimated in five cases because the information needed for this purpose was unavailable; hence, the calculation of value-based price with respect to the device could be performed in 42 cases.

In our comparisons between real prices and value-based prices for the above-mentioned 42 devices (Figure 3), real prices were appreciably lower than value-based prices in 36 cases (86%) and were similar (±15%) in three cases. Three devices showed a real price substantially greater than the value-based price (Perceval for severe aortic stenosis, Yttrium-90 microspheres for hepatocellular carcinoma, and the Argus II retinal prosthesis system). These three devices deserve to be examined in more detail.

Perceval is a suture-less valve indicated in severe aortic stenosis. The profile of its price (real price, 3107 €; value-based price, 2155 €; ratio, 1.44) is mainly guided by the low incidence of the device cost (irrespective of its nature of real price or value-based price) over the overall cost for treating these patients.

Yttrium-90 microspheres are used for trans-arterial radioembolization in locally advanced and inoperable hepatocellular carcinoma as an alternative to sorafenib; the microsphere treatment was not found to be a cost-effective option at the usually accepted WTP thresholds. The ICER was 2,863,167 € per QALY gained; this unfavorable result was mainly guided by the gain QALYs, which was minimal. Regarding prices, the real price is about twice the value-based price (ratio, 2.07).

Argus II is a retinal prosthesis system for advanced retinitis pigmentosa. This device helps people see better and is generally safe to use. The device is costly (around 180,000 Canadian$), but because retinitis pigmentosa is rare, the budget impact of publicly funding this device in Canada is less than 1 million $ per year (assuming four implants per year per patient). Regarding prices, also for this device the real price is about twice the value-based price (ratio, 2.15). Table 2 shows detailed information about real price, value-based price, and their ratio for each device analyzed in Figure 3.

**Table 2:** Detailed information for each device about real price, value-based price, and their ratio.

DEVICE	Real price in €	Value-based price in €	Ratio real vs. value-based
OPTOGENERAPY (Optoferon)	30395	negative value	NR
Triangular titanium implant for sacroiliac joint fusion	2886	39140	0.074
SU-AVR (sutureless aortic valve replacement)	6000	80971	0.074
WavelinQ (incident patients)	2544	30943	0.082
AUS (artificial urinary sphincter)	6344	61353	0.103
AUS (artificial urinary sphincter)	6344	61353	0.103
ILUVIEN (Fac implant vs DEXA implant)	6729	38812	0.173
Y-90 microspheres	6664	37866	0.176
SAPIEN 3	15419	81561	0.189
IStent inject device	1300	5766	0.225
SAPIEN 3	24078	89711	0.268
Pathello-femoral arthroplasty (Avon implant by Stryker Orthopaedics, Mahwah, New Jersey, USA).	1603	5921	0.271
Tyrx	599	1889	0.317
SAPIEN 3	31794	95076	0.334
Mitraclip	13745	40344	0.341
Barostim neo	21000	59215	0.355
Biodesign Surgisis (Cook)	650	1572	0.413
DEX-700, dexa- methasone implant (implant + current practice vs current practice alone),	725	1698	0.427
InSpace	15000	34704	0.432
Nexplanon (etonogestrel 68 mg implant)	276	599	0.460
iStent Inject trabecular bypass stent (TBS) device	1249	2569	0.486
ILUVIEN (Fac implant vs usual care)	6649	13591	0.489
SAPIEN 3	24078	46941	0.513
SAPIEN 3	24078	45323	0.531
SAPIEN 3	18658	32417	0.576
InSpace	15000	25585	0.586
Amplatzer	3735	6034	0.619
SAPIEN 3	18658	30096	0.620
OZURDEX	1075	1628	0.660
aMACE	8419	12684	0.664
ILUVIEN (Fac implant vs usual care)	6729	9586	0.702
HEARTMATE 3	66640	91884	0.725
InSpace	15000	17478	0.858
TAVR (transcatheter aortic valve replacement)	17911	20338	0.881
Propel (bioabsorbable corticosteroid-eluting sinus implant)	483	541	0.893
FENIX TORAX	3332	3459	0.963
Reducer	7000	6566	1.066
TAVR (transcatheter aortic valve replacement)	16419	14251	1.152
OPRA (Osteointegrated Prostheses for the Rehabilitation of Amputees)	48139	41347	1.150
Perceval	32500	22539	1.442
Yttrium-90 resin microspheres for transarterial radioembolization	32534	15715	2.070
Argus II	134222	62467	2.149

Discussion

The main finding deriving from our study is that the great majority of economic analyses focused on medical devices show a favorable cost-effectiveness profile, while an unfavorable profile was found in a small minority of cases (3 out of 42, 7.1%). This is a quite unexpected result for which no straightforward explanation can be proposed, but some plausible hypotheses can however be considered. Firstly, it is well known that a remarkable publication bias affects pharmacoeconomic and cost-effectiveness research [6]. Since economic analyses reporting favorable results are more likely to be published than those reporting unfavorable results, our study might simply reflect this well-known tendency. Another hypothesis is that, in the pharmaceutical market, more economic research is being carried out than in the field of devices, and so more efforts are made to claim high prices for medicines than for devices.

Regarding the areas where value-based price deserves to be applied, they include especially the case of innovative devices [52], where innovation can be managed according to recent definitions [9]; also tenders are suitable for the application of value-based pricing [53]. In both cases, value-based prices can be useful either to guide the first purchase of a new device or to determine the starting price of a tender lot.

Our study likely reflects the pros and the cons that are typical to any research conducted in an unexplored area. In the first place, our results suggest the need for a wider application of this type of analysis. On the other hand, since this is a preliminary report, other studies hopefully will follow, likely designed in a different way and more based on real data than on literature data.

In our previous article [12], we emphasized a few points, that are fully confirmed by the present investigation and that we repropose with nearly the same words:

Point 1: When the literature search of cost-effectiveness data is successful, obtaining the value-based price proves to be an easy task.

Point 2: The method proposed herein for value-based price estimation is an approximate one. On this premise, when the reference cost-effectiveness study has been conducted in a foreign country with a health-care system similar to ours, the various items of health-care cost generally remain valid (apart from the mandatory conversion from one currency into another). Much caution is instead needed when the health-care system is substantially different; in such a case, clinical data likely remain valid, but the information on costs needs to be entirely reassessed.

Point 3: The availability of adequate literature data is a critical issue influencing the successful application of value-based pricing. When adequate data are not available, how to fill this gap in information remains an open question.

## Conclusions

To summarize the current state of the art concerning the methods for the governance of medical devices, the application of the principles of cost effectiveness is increasingly considered a mandatory objective. There are two main fields of application. Firstly, when tenders are concerned, cost effectiveness can be used both to set the starting price of the various lots and to adjudicate the winner of each lot through a balanced evaluation of costs and benefits; in this balance, the winner is not the product that offers the lowest price, but the product that offers the best ratio between all sources of cost (including the price offered), and all benefits; it should be stressed that, in the evaluation of benefits, all clinical parameters (e.g., the outcomes) must be expressed in monetary units, and reference must explicitly be made to the societal WTP threshold recognized locally. Secondly, also in the assessment of each innovative product, cost-effectiveness is applied but in a different fashion: on the premise that a WTP is recognized, the value-based price of the innovative product needs to be estimated, and a comparison must be made between the price offered and the value-based price calculated separately. In addition, the same analysis can be made though in the opposite direction; the cost effectiveness ratio of the product is calculated, and this ratio is compared with the WTP threshold recognized by the society. This comparison permits to conclude that the economic profile of the new product is favorable, when the ratio remains below the threshold, and instead is unfavorable when the ratio exceeds the threshold. in more general terms, the need to interpret more accurately the available literature is another important point; this implies to use specific investigation methods (e.g., the gap analysis) to understand why the area of devices is so less advanced than that of medicines, and to better differentiate the clinical evidence that emerges from the real world from that generated within formal clinical trials.

All in all, in this framework, the application of cost-effectiveness is important especially for advanced medical devices and therefore needs to be promoted more and more. Regarding the value-based price, when the available data allow for their estimation, the practical relevance of these estimates is extremely high, mainly because its absence would imply to purchase these products with no objective pricing criterion. On the other hand, one should be aware of the unsolved drawbacks of these procurement methods, e.g. when cost data must be transferred from one country to another.

## References

[1] Claxton K, Briggs A, Buxton MJ, Culyer AJ, McCabe C, Walker S, Sculpher MJ (2008). Value based pricing for NHS drugs: an opportunity not to be missed?. BMJ.

[2] Jommi C, Armeni P, Costa F, Bertolani A, Otto M (2020). Implementation of value-based pricing for medicines. Clin Ther.

[3] Kaltenboeck A, Bach PB (2018). Value-based pricing for drugs: theme and variations. JAMA.

[4] Garner S, Rintoul A, Hill SR (2018). Value-based pricing: L'Enfant terrible?. PharmacoEconomics.

[5] Shiroiwa T (2020). Cost-effectiveness evaluation for pricing medicines and devices: a new value-based price adjustment system in Japan. Int J Technol Assess Health Care.

[6] Bell CM, Urbach DR, Ray JG, Bayoumi A, Rosen AB, Greenberg D, Neumann PJ (2006). Bias in published cost effectiveness studies: systematic review. BMJ.

[7] World Health Organization (2020). WHO Guideline on Country Pharmaceutical Pricing Policies, Second Edition. https://apps.who.int/iris/bitstream/handle/10665/335692/9789240011878-eng.pdf.

[8] Prieto-Pinto L, Garzón-Orjuela N, Lasalvia P, Castañeda-Cardona C, Rosselli D (2020). International experience in therapeutic value and value-based pricing: a rapid review of the literature. Value Health Reg Issues.

[9] Messori A, Trippoli S, Bartoli L, Marinai C (2023). Defining innovativeness of high-technology medical devices in an Italian region. Eur J Hosp Pharm.

[10] Rahmani K, Karimi S, Rezayatmand R, Raeisi AR (2021). Value-based procurement for medical devices: a scoping review. Med J Islam Repub Iran.

[11] Trippoli S, Messori A, Borselli G, Autieri F, Mamone D, Marinai C (2022). Relationship between price and diagnosis-related group tariff for medical devices assessed by a regional health technology assessment committee. Cureus.

[12] Messori A, Trippoli S (2022). Estimation of value-based price for five high-technology medical devices approved by a regional health technology assessment committee in Italy. Cureus.

[13] Regione Toscana (2023). Regione Toscana. Prodotti HTA. https://www.regione.toscana.it/-/prodotti-hta.

[14] Allou A, Baschet L, Sabourin C, Montalscot G, Lorgis L, Iriart X (2022). Cost-effectiveness analysis of patent foramen ovale closure with Amplatzer plus medical therapy compared to medical therapy in patients with a history of stroke in France. J Cardiol.

[15] Borisenko O, Müller-Ehmsen J, Lindenfeld J, Rafflenbeul E, Hamm C (2018). An early analysis of cost-utility of baroreflex activation therapy in advanced chronic heart failure in Germany. BMC Cardiovasc Disord.

[16] Lim HS, Shaw S, Carter AW, Jayawardana S, Mossialos E, Mehra MR (2022). A clinical and cost-effectiveness analysis of the HeartMate 3 left ventricular assist device for transplant-ineligible patients: a United Kingdom perspective. J Heart Lung Transplant.

[17] Chew DS, Manns B, Miller RJ, Sharma N, Exner DV (2017). Economic evaluation of left ventricular assist devices for patients with end stage heart failure who are ineligible for cardiac transplantation. Can J Cardiol.

[18] Shore J, Russell J, Frankenstein L, Candolfi P, Green M (2020). An analysis of the cost-effectiveness of transcatheter mitral valve repair for people with secondary mitral valve regurgitation in the UK. J Med Econ.

[19] Desser AS, Arentz-Hansen H, Fagerlund BF, Harboe I, Lauvrak V (2017). Sutureless Aortic Valve Replacement for Treatment of Severe Aortic Stenosis: A Single Technology Assessment of Perceval Sutureless Aortic Valve. https://fhi.brage.unit.no/fhi-xmlui/bitstream/handle/11250/2457904/Desser_2017_Sut.pdf?sequence=1.

[20] Villemoes MK, Lindholt JS, Houlind KC (2018). Cost-effectiveness evaluation of heparin coated versus standard graft for bypass surgery in peripheral artery disease alongside a randomised controlled trial. Eur J Vasc Endovasc Surg.

[21] Gallone G, Armeni P, Verheye S (2020). Cost-effectiveness of the coronary sinus Reducer and its impact on the healthcare burden of refractory angina patients. Eur Heart J Qual Care Clin Outcomes.

[22] Goodall G, Lamotte M, Ramos M, Maunoury F, Pejchalova B, de Pouvourville G (2019). Cost-effectiveness analysis of the SAPIEN 3 TAVI valve compared with surgery in intermediate-risk patients. J Med Econ.

[23] Mennini FS, Meucci F, Pesarini G (2022). Cost-effectiveness of transcatheter aortic valve implantation versus surgical aortic valve replacement in low surgical risk aortic stenosis patients. Int J Cardiol.

[24] Pinar E, García de Lara J, Hurtado J (2022). Cost-effectiveness analysis of the SAPIEN 3 transcatheter aortic valve implant in patients with symptomatic severe aortic stenosis. Rev Esp Cardiol (Engl Ed).

[25] Gilard M, Eltchaninoff H, Iung B (2022). Cost-effectiveness analysis of SAPIEN 3 transcatheter aortic valve implantation procedure compared with surgery in patients with severe aortic stenosis at low risk of surgical mortality in France. Value Health.

[26] Tarride JE, Luong T, Goodall G, Burke N, Blackhouse G (2019). A Canadian cost-effectiveness analysis of SAPIEN 3 transcatheter aortic valve implantation compared with surgery, in intermediate and high-risk severe aortic stenosis patients. Clinicoecon Outcomes Res.

[27] Povero M, Miceli A, Pradelli L, Ferrarini M, Pinciroli M, Glauber M (2018). Cost-utility of surgical sutureless bioprostheses vs TAVI in aortic valve replacement for patients at intermediate and high surgical risk. Clinicoecon Outcomes Res.

[28] Tam DY, Hughes A, Wijeysundera HC, Fremes SE (2018). Cost-effectiveness of self-expandable transcatheter aortic valves in intermediate-risk patients. Ann Thorac Surg.

[29] Kay G, Eby EL, Brown B (2018). Cost-effectiveness of TYRX absorbable antibacterial envelope for prevention of cardiovascular implantable electronic device infection. J Med Econ.

[30] Lundqvist M, Levin LÅ (2020). Cost-effectiveness of the use of Gold Anchor™ markers in prostate cancer. Cureus.

[31] Muszbek N, Remak E, Evans R (2021). Cost-utility analysis of selective internal radiation therapy with Y-90 resin microspheres in hepatocellular carcinoma. Future Oncol.

[32] Zarca K, Mimouni M, Pereira H, Chatellier G, Vilgrain V, Durand-Zaleski I (2021). Cost-utility analysis of transarterial radioembolization with Yttrium-90 resin microspheres compared with sorafenib in locally advanced and inoperable hepatocellular carcinoma. Clin Ther.

[33] Ahmed II, Podbielski DW, Patel V, Falvey H, Murray J, Botteman M, Goeree R (2020). A Canadian cost-utility analysis of 2 trabecular microbypass stents at time of cataract surgery in patients with mild to moderate open-angle glaucoma. Ophthalmol Glaucoma.

[34] Fea AM, Cattel F, Gandolfi S, Buseghin G, Furneri G, Costagliola C (2021). Cost-utility analysis of trabecular micro-bypass stents (TBS) in patients with mild-to-moderate open-angle Glaucoma in Italy. BMC Health Serv Res.

[35] Health Quality Ontario (2017). Retinal prosthesis system for advanced retinitis pigmentosa: a health technology assessment update. Ont Health Technol Assess Ser.

[36] Pesonen M, Kankaanpää E, Vottonen P (2021). Cost-effectiveness of dexamethasone and triamcinolone for the treatment of diabetic macular oedema in Finland: a Markov-model. Acta Ophthalmol.

[37] Pochopien M, Beiderbeck A, McEwan P, Zur R, Toumi M, Aballéa S (2019). Cost-effectiveness of fluocinolone acetonide implant (ILUVIEN®) in UK patients with chronic diabetic macular oedema considered insufficiently responsive to available therapies. BMC Health Serv Res.

[38] Squires H, Bermejo I, Poku EN (2019). Dexamethasone implant for non-infectious uveitis: is it cost-effective?. Br J Ophthalmol.

[39] Hansson E, Hagberg K, Cawson M, Brodtkorb TH (2018). Patients with unilateral transfemoral amputation treated with a percutaneous osseointegrated prosthesis: a cost-effectiveness analysis. Bone Joint J.

[40] Castagna A, Garofalo R, Maman E, Gray AC, Brooks EA (2019). Comparative cost-effectiveness analysis of the subacromial spacer for irreparable and massive rotator cuff tears. Int Orthop.

[41] Fredborg C, Odgaard A, Sørensen J (2020). Patellofemoral arthroplasty is cheaper and more effective in the short term than total knee arthroplasty for isolated patellofemoral osteoarthritis: cost-effectiveness analysis based on a randomized trial. Bone Joint J.

[42] Blissett DB, Blissett RS, Ede MP, Stott PM, Cher DJ, Reckling WC (2021). Minimally invasive sacroiliac joint fusion with triangular titanium implants: cost-utility analysis from NHS perspective. Pharmacoecon Open.

[43] Tack P, Victor J, Gemmel P, Annemans L (2021). Do custom 3D-printed revision acetabular implants provide enough value to justify the additional costs? The health-economic comparison of a new porous 3D-printed hip implant for revision arthroplasty of Paprosky type 3B acetabular defects and its closest alternative. Orthop Traumatol Surg Res.

[44] Jayne DG, Scholefield J, Tolan D (2021). A multicenter randomized controlled trial comparing safety, efficacy, and cost-effectiveness of the surgisis anal fistula plug versus surgeon's preference for transsphincteric fistula-in-ano: the FIAT trial. Ann Surg.

[45] Jayne DG, Williams AE, Corrigan N (2021). Sacral nerve stimulation versus the magnetic sphincter augmentation device for adult faecal incontinence: the SaFaRI RCT. Health Technol Assess.

[46] Shamout S, Nazha S, Dragomir A, Campeau L (2018). A cost-utility analysis of artificial urinary sphincter versus AdVance male sling in post prostatectomy stress urinary incontinence: a publicly funded health care perspective. Neurourol Urodyn.

[47] Visser LA, Folcher M, Delgado Simao C, Gutierrez Arechederra B, Escudero E, Uyl-de Groot CA, Redekop WK (2022). The potential cost-effectiveness of a cell-based bioelectronic implantable device delivering interferon-β1a therapy versus injectable interferon-β1a treatment in relapsing-remitting multiple sclerosis. PharmacoEconomics.

[48] Pietzsch JB, Richter AK, Randerath W (2019). Clinical and economic benefits of upper airway stimulation for obstructive sleep apnea in a European setting. Respiration.

[49] Javanbakht M, Saleh H, Hemami MR, Branagan-Harris M, Boiano M (2020). A corticosteroid-eluting sinus implant following endoscopic sinus surgery for chronic rhinosinusitis: a UK-based cost-effectiveness analysis. Pharmacoecon Open.

[50] CADTH CADTH (2020). Pharmacoeconomic Report: Etonogestrel Extended-Release Subdermal Implant (Nexplanon). J Thorac Cardiovasc Surg.

[51] Rognoni C, Tozzi M, Tarricone R (2021). Endovascular versus surgical creation of arteriovenous fistula in hemodialysis patients: cost-effectiveness and budget impact analyses. J Vasc Access.

[52] Messori A, Trippoli S, Fadda V, Romeo MR (2023). Managing tenders in the procurement of advanced medical devices: an original model based on the net monetary benefit combined with three clinical endpoints. Cureus.

[53] Messori A, Trippoli S, Caccese E, Marinai C (2020). Tenders for the procurement of medical devices: adapting cost-effectiveness rules to the requirements of the European public procurement directive. Ther Innov Regul Sci.

